# Effect of Heat Treatment Process on Microstructure and Fatigue Behavior of a Nickel-Base Superalloy

**DOI:** 10.3390/ma8095299

**Published:** 2015-09-16

**Authors:** Peng Zhang, Qiang Zhu, Gang Chen, Heyong Qin, Chuanjie Wang

**Affiliations:** 1 School of Materials Science and Engineering, Harbin Institute of Technology at Weihai, Weihai 264209, China; E-Mails: pzhang@hit.edu.cn (P.Z.); mse_zhu@yeah.net (Q.Z.); mse_chen@yeah.net (G.C.); 2 Central Iron & Steel Research Institute, Beijing 100081, China; E-Mail: mse_qin@yeah.net

**Keywords:** heat treatment, microstructure, fatigue behavior, fatigue life model, fracture mechanism

## Abstract

The study of fatigue behaviors for nickel-base superalloys is very significant because fatigue damage results in serious consequences. In this paper, two kinds of heat treatment procedures (Pro.I and Pro.II) were taken to investigate the effect of heat treatment on microstructures and fatigue behaviors of a nickel-base superalloy. Fatigue behaviors were studied through total strain controlled mode at 650 °C. Manson-Coffin relationship and three-parameter power function were used to predict fatigue life. A good link between the cyclic/fatigue behavior and microscopic studies was established. The cyclic deformation mechanism and fatigue mechanism were discussed. The results show that the fatigue resistance significantly drops with the increase of total strain amplitudes. Manson-Coffin relationship can well predict the fatigue life for total strain amplitude from 0.5% to 0.8%. The fatigue resistance is related with heat treatment procedures. The fatigue resistance performance of Pro.I is better than that of Pro.II. The cyclic stress response behaviors are closely related to the changes of the strain amplitudes. The peak stress of the alloy gradually increases with the increase of total strain amplitudes. The main fracture mechanism is inhomogeneous deformation and the different interactions between dislocations and γ′ precipitates.

## 1. Introduction

Nickel-base superalloys have become critical materials for high temperature components of gas-turbine engines due to high temperature strength, oxidation resistance, excellent creep resistance, excellent fatigue resistance at elevated temperature [1,2,3]. Turbine disk, an important high temperature component of gas-turbine engines, withstands effects of high temperatures and alternating loading in actual service conditions. In this case, strain-controlled high temperature low cycle fatigue (LCF) damage occurs for turbine disk. Fatigue damage seriously affects the persistent and normal use of high temperature components. In addition, fatigue damage reduces their service lives and leads to catastrophic consequences [4,5]. Thus, the study of fatigue behaviors for nickel-base superalloys is necessary and significant. Numerous studies have been carried out about the effects of cyclic frequency, inclusions, microstructure, strain range, mean stress, pre-deformation, environment and testing temperature on fatigue behaviors of nickel-base superalloys [6,7,8,9,10].

The alloy studied is a precipitation-hardened high nickel superalloy, which is used extensively in the gas-turbine engines. The effect of high nickel is to improve the plasticity and ductility of the superalloy. In order to get a perfect overall performance of turbine disks, especially excellent fatigue resistance, heat treatment process is an important factor in addition to its composition and appropriate manufacturing process. The appropriate heat treatment process can give full play to the potential of alloys and ensure its safe and reliable work. The effects of heat treatment process on microstructure and fatigue behavior of nickel-base superalloys have been less investigated so far. Du *et al.* [11] investigated the effects of the solution and aging treatment on the microstructure of GH4169 superalloy. James and Mills [12] studied the fatigue-crack growth behavior of Alloy 718 at different test temperatures. Electron fractographic examination revealed that operative crack growth mechanisms were dependent on heat treatment, heat-to-heat variations, testing temperature and stress-intensity level of crack tip. In this paper, the effects of heat treatment process on microstructure and fatigue behavior of the alloy were simultaneously investigated. Two kinds of heat treatment procedures were taken for the nickel-base superalloy. Then fatigue tests were conducted for the specimens selected from the heat treatment procedures. It is of great significance to study the effect of different solution treatments on microstructures and fatigue behaviors of the superalloy. As for gas-turbine engine turbine disks, the actual service temperature is about 650 °C. LCF behaviors of the alloy were studied at 650 °C under condition of total strain-controlled mode. The effect of strain amplitudes on cycle mechanical response was studied. Moreover, the microstructures of different heat treatment procedures after fatigue test were investigated. A good link between the cyclic/fatigue behavior and microscopic studies was established. It would be much meaningful to improve the production of nickel-base superalloys and fatigue life of turbine disks through the study of LCF behaviors for the nickel-base superalloy by different heat treatment procedures.

## 2. Experimental Section

The nickel-base superalloy was smelted by vacuum induction furnace and re-melted by vacuum consumable electrode melting furnace. In order to obtain uniformity of the grain size, the nickel-base superalloy was forged at 1100 °C. The chemical composition (wt %) of the alloy is as follows: C 0.042, Cr 14.50, Mo 3.18, Al 1.70, Ti 2.68, Fe < 0.10, Nb 2.02, and Ni the rest.

### 2.1. Heat Treatment Procedure

In order to improve the mechanical properties of the alloy and eliminate residual stress, especially to investigate the effect of heat treatment process on LCF behavior, two kinds of heat treatment procedures were taken, which are as follows:

ProcedureI (Pro.I): 1050 °C/8 h, AC + 1000 °C/4 h, AC + 775 °C/16 h, AC + 700 °C/16 h, AC
ProcedureII (Pro.II): 1100 °C/8 h, AC + 1000 °C/4 h, AC + 775 °C/16 h, AC + 700 °C/16 h, AC

Where, AC means air cooling. Heat treatment procedures are four stages. The first stage is solution treatment. For example, the stage “1050 °C/8 h, AC” means that the specimens were heated from room temperature to 1050 °C and heat preservation time was 8 h at a constant temperature of 1050 °C. Then the specimens were air-cooled to room temperature. The second stage (1000 °C/4 h, AC) is intermediate heat treatment and the last two stages (775 °C/16 h, AC + 700 °C/16 h, AC) are aging treatment.

### 2.2. Fatigue Tests

The standard of fatigue tests is referred to ISO 1099:2006 (Metallic materials-Fatigue testing-Axial-force-controlled method) [13]. The fatigue specimens selected from heat-treated specimens were machined into 6.350 mm in diameter and 16 mm in gauge length. LCF tests were carried out on MTS 810 fatigue testing machine (MTS Systems Corporation, Eden Prairie, MN, USA) in air. Experimental program was total axial strain-controlled from 0.3% to 0.8% at constant temperature of 650 °C. The strain ratio was *R* = −1 and the loading frequency was 0.50 Hz. The fatigue specimens were mechanical grinded, polished and chemical etched so as to observe grain morphology of the nickel-base superalloy by Olympus microscope. The precipitates of the nickel-base superalloy after heat treatment were observed by Quanta 200FEG scanning electron microscopy (SEM, FEI Company, Hillsboro, OR, USA). In order to investigate the fracture mechanism of the alloy, the fracture morphology of fatigue failure specimens was observed by SEM and the dislocation characteristics were observed by Tecnai G2 F30 transmission electron microscopy (TEM, FEI Company, Hillsboro, OR, USA). Specimens for TEM were obtained from thin slices (500 µm in thickness) at a distance of 1 mm away from the fracture surfaces of the failed specimens. Thin slices were ground to about 50 μm, and then prepared for twin-jet electrolytic thinning.

## 3. Cyclic Stress Response Behavior and Deformation Mechanism

### 3.1. Grain Morphology and Precipitates

**Figure 1:**
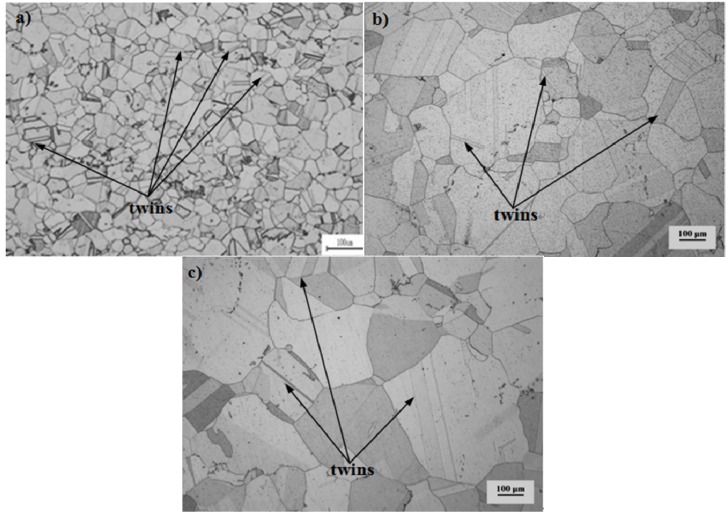
The grain morphology of the nickel-base superalloy. (**a**) the original grain; (**b**) Pro.I; (**c**) Pro.II.

**Figure 2:**
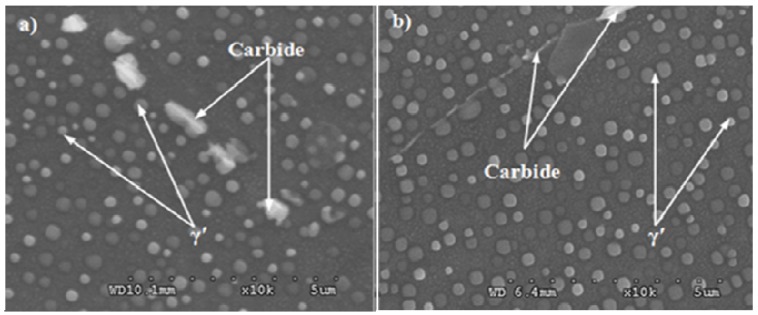
The precipitates of the nickel-base superalloy after heat treatment. (**a**) Pro.I; (**b**) Pro.II.

The purpose of solution treatment is to dissolve the original reinforcing phases, to dissolve the irrational distributed secondary carbide and boride phases on grain boundary, to obtain the desired grain size and to eliminate residual stresses. The purpose of the aging treatment is to precipitate evenly fine dispersive distributed γ′ phases, at the same time to precipitate the complex carbide and boride phases on grain boundary. Turbine disks will possess a perfect overall performance after heat treatment. Figure 1 shows the grain morphology of the alloy. It can be observed that the average size of the original grain is the smallest and average grain size of Pro.I is much smaller than that of Pro.II. In addition, many twins exist in the original grain and some twins still exist after heat treatment. The grain sizes are measured by intersection point method. The average sizes of original grain, Pro.I and Pro.II are 31.4 μm, 70.7 μm and 144.3 μm, respectively. The precipitates of the nickel-base superalloy after heat treatment are shown in Figure 2. The incipient melting temperature of γ′ phase for the alloy is 1050 °C and fully melting temperature of γ′ phase is 1080 °C. As for Pro.I, solution temperature is lower than fully melting temperature of γ′ phase. As for Pro.II, the solution temperature is higher than fully melting temperature of γ′ phase. It can be observed from Figure 2 that the distribution of γ′ phases for Pro.II is more uniform than that for Pro.I. It shows that undissolved γ′ phases in the solution treatment process hinder grain growth. The purpose of the intermediate heat treatment is to make small γ′ phases formed during cooling after solution treatment grow up as ideal large γ′ phases and to re-precipitate small γ′ phases. There are some large carbide particles at the grain boundaries.

### 3.2. Fatigue Property and Fatigue Life Model

The fatigue test results under different heat treatment procedures are shown in Table 1, where, Δε_t_/2 is the total strain, Δε_e_/2 is the elastic strain, Δε_p_/2 is the peak plastic strain, and 2*N*_f_ is the reverse number of fatigue cycles. It can be found that fatigue resistance significantly drops with the increase of strain amplitudes under the condition of the same heat treatment procedure. It can be observed that the fatigue life of Pro.I is higher than that of Pro.II at the same total strain amplitude, which indicates that the heat treatments play an important role in fatigue behavior and the fatigue resistance of Pro.I is better than that of Pro.II.

**Table 1:** Fatigue test results under different heat treatment procedures.

Heat Treatment	Δ ε_t_/2 (%)	Δ ε_e_/2 (%)	Δ ε_p_/2 (%)	2*N*_f_
Pro.I	0.3	0.297	0.003	68,468
	0.4	0.378	0.022	8094
	0.5	0.413	0.087	1746
	0.6	0.473	0.127	768
	0.7	0.489	0.211	282
	0.8	0.51	0.29	174
Pro.II	0.3	0.297	0.003	52,032
	0.4	0.368	0.032	4528
	0.5	0.414	0.086	1622
	0.6	0.466	0.134	648
	0.7	0.491	0.209	242
	0.8	0.508	0.292	148

For strain-controlled LCF, total strain amplitude includes elastic strain and plastic strain. The Manson-Coffin relationship can be used to describe the strain-controlled LCF behavior. According to Manson-Coffin relationship and Basqin relationship, the relationship of the elastic, plastic and total strain ranges is expressed as follow [14]:

(1) $$\frac{\Delta {\text{ε}}_{t}}{2}=\frac{\Delta {\text{ε}}_{e}}{2}+\frac{\Delta {\text{ε}}_{p}}{2}=\frac{{{\text{σ}}^{/}}_{\text{f}}}{E}{\left(2N_{\text{f}}\right)}^{b}+{{\text{ε}}^{/}}_{\text{f}}{\left(2N_{\text{f}}\right)}^{c}$$

where σ′_f_ is the fatigue strength coefficient; ε′_f_ is the fatigue ductility coefficient; *b* is the fatigue strength exponent, *c* is the fatigue ductility exponent; *E* is Young’s modulus.

**Figure 3:**
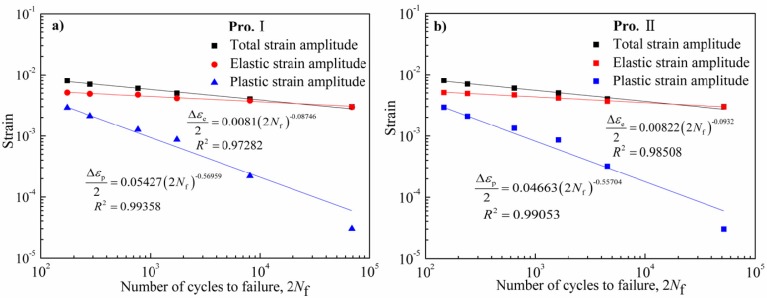
Total; elastic and plastic strain range *vs.* number of cycles to failure of the nickel-base alloy. (**a**) Pro.I; (**b**) Pro.II.

Figure 3 shows the relationship of the elastic, plastic and total strain amplitudes *versus* number of cycles to failure for Pro.I and Pro.II. The relationship curves of the elastic and plastic strain amplitudes *versus* number of cycles to failure were fitted according to Equation (1). Then parameters dependent on LCF of Pro.I and Pro.II can be obtained, as shown in Figure 3. The corresponding Manson-Coffin models are shown as follows:

(2) $$\text{Pro.I: }\frac{\Delta {\text{ε}}_{t}}{2}=0.0081{\left(2N_{\text{f}}\right)}^{-0.08746}+0.05427{\left(2N_{\text{f}}\right)}^{-0.56959}$$

(3) $$\text{Pro.II: }\frac{\Delta {\text{ε}}_{t}}{2}=0.00822{\left(2N_{\text{f}}\right)}^{-0.0932}+0.04663{\left(2N_{\text{f}}\right)}^{-0.55704}$$

The relationship of three-parameter power function can also be used to describe the strain-controlled LCF behavior. The relationship of total strain range to fatigue life is expressed by Equation (4) as follows:

(4) $$N_{f}{\left(\Delta {\text{ε}}_{t}-\Delta {\text{ε}}_{0}\right)}^{m}=c$$

whereΔε_0_, *m* and *c* are material constants. Fatigue limits of alloys are considered in the relationship.

**Figure 4:**
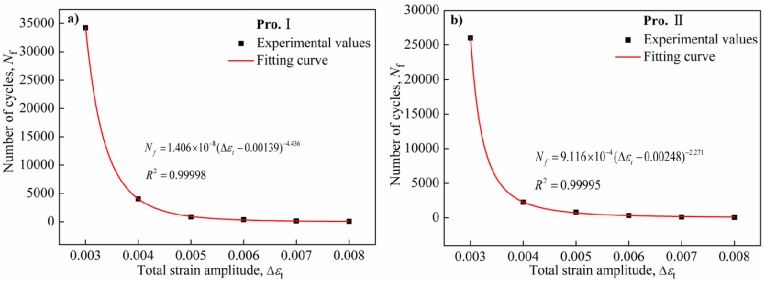
The reciprocal of cycles to failure *versus* the total strain ranges of the nickel-base alloy. (**a**) Pro.I; (**b**) Pro.II.

The relationship curves of the cycles to failure *versus* the total strain ranges for Pro.I and Pro.II are depicted in Figure 4. The relationship curves of the total strain ranges *versus* number of cycles to failure were fitted according to Equation (4). The corresponding fatigue life models are shown as follows:

(5) $$\text{Pro.I: }N_{f}=1.40603\times 10^{-8}{\left(\Delta \varepsilon_{t}-0.00139\right)}^{-4.43574}$$

(6) $$\text{Pro.II: }N_{f}=9.11575\times 10^{-4}{\left(\Delta \varepsilon_{t}-0.00248\right)}^{-2.27067}$$

**Figure 5:**
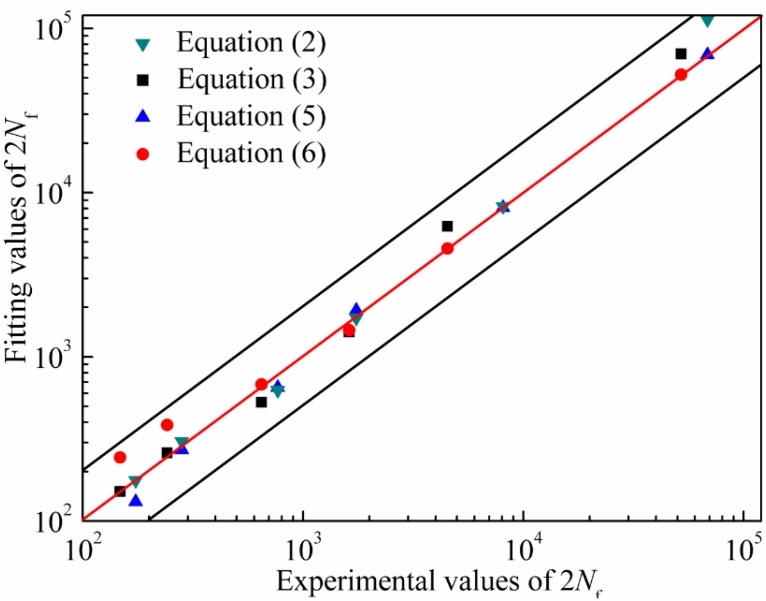
The comparisons of the experimental values and the fitting values.

Equations (2) and (3) and Equations (5) and (6) can be used to predict the fatigue life of the alloy at 650 °C as long as the total strain amplitudes are known. The comparisons of experimental values and predicted values of 2 *N*_f_ are shown in Figure 5. The predicted effect is perfect when the fatigue life predicted points fall on the red line. It can be found that fatigue life predicted points fall basically within two fold safety factor specified dispersing band. As for Manson-Coffin relationship, the predicted effect is in good agreement with total strain amplitudes from 0.5% to 0.8% and poor for total strain amplitudes from 0.3% to 0.4%. However, as for three-parameter power function, the predicted effect is in good agreement with total strain amplitudes from 0.3% to 0.6% and poor for total strain amplitudes from 0.7% to 0.8%. The conclusion that the predicted effect of Manson-Coffin relationship is more accurate than that of three-parameter power function for high total strain amplitude section can be drawn.On the contrary, the predicted effect of three-parameter power function is more accurate than that of Manson-Coffin relationship for low total strain amplitude section. The relevance of experimental values and predicted values is used to evaluate the predicted effect of fatigue life model in the engineering. In order to evaluate fatigue life prediction method for above two kinds of fatigue life models, an error analysis method is adopted. The parameters of error analysis method are as follows [15]:

(7) $$Err=\log(N_{p}/N_{e})$$

(8) $$\bar{E}=\frac{1}{n}\sum_{1}^{n}\left|Err\right|$$

where $N_{p}$ is predicted life; $N_{e}$ is experimental life; *Err* is error and $\bar{E}$ is mean error.

The error analysis of the life data is presented in Table 2. Its results are consistent with the analysis of Figure 5. It can be inferred that the predicted results are consistent with experimental results. On the whole, Manson-Coffin relationship can well predict the fatigue life of the nickel-base alloy studied in this paper due to the smaller error.

**Table 2:** Error analysis of life prediction (%).

Heat Treatment	Manson-Coffin	Three-Parameter Power Function
Pro.I	2.83	2.79
Pro. II	2.65	6.61

### 3.3. Cyclic Stress Response Behavior

**Figure 6:**
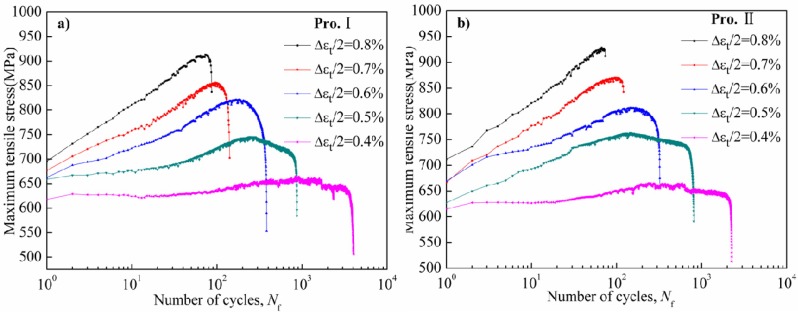
Cyclic stress response curve of the nickel-base alloy at 650 °C. (**a**) Pro.I; (**b**) Pro.II.

Cyclic stress response behavior includes cyclic hardening, softening and stability under different experimental conditions. The fundamental reason of cyclic stress response depends on the microstructure of the alloy, which includes different dislocation motions and the interactions between dislocations and γ' phases. The changes in tensile peak stress *versus* logarithm of number of cycles of Pro.I and Pro.II under total strain amplitudes of 0.4% to 0.8% are plotted in Figure 6. It can be seen that stress amplitude and cyclic stress response behavior are closely related to the strain amplitudes.

As is shown in Figure 6, the peak stress of the alloy gradually increases with the increase of total strain amplitudes in the process of LCF, from 659 MPa under low strain amplitude of 0.4% to 904 MPa under high strain amplitude of 0.8% for Pro.I and from 661 MPa under low strain amplitude of 0.4% to 910 MPa under high strain amplitude of 0.8% for Pro.II.

It can be seen in Figure 6 that cyclic hardening, softening and stability behaviors simultaneously exist in the LCF process for Pro. I and Pro. II. In terms of Pro. I, under total strain amplitudes of 0.4% and 0.5%, the alloy exhibits a continuous cyclic stability response and then cyclic hardening, followed cyclic softening till failure. Under total strain amplitudes of 0.6%, 0.7% and 0.8%, the alloy exhibits a continuous cyclic hardening response till failure and the hardening capability increases with the increase of strain amplitudes. In terms of Pro. II, the change trend of cyclic stress response curve is same with that of Pro. I except total strain amplitude of 0.5%. Under total strain amplitude of 0.5%, the alloy exhibits a continuous cyclic hardening response and then cyclic softening response till failure.

Before the final failure fracture, cyclic stress response behavior is characterized by the condition of rapid decline because of the formation of macro crack and the subsequent crack propagation till failure. It can be seen that the number of cycles for rapid decline before the final failure fracture of Pro. I are more than that of Pro. II under high strain amplitudes from 0.6% to 0.8%. It can be inferred that Pro. II causes more remarkable harming. It can also indicate that the heat treatments possess significant effect on fatigue behavior and the fatigue resistance of Pro. I is better than that of Pro. II.

### 3.4. Fatigue Fracture Morphology and Fracture Mechanism

**Figure 7:**
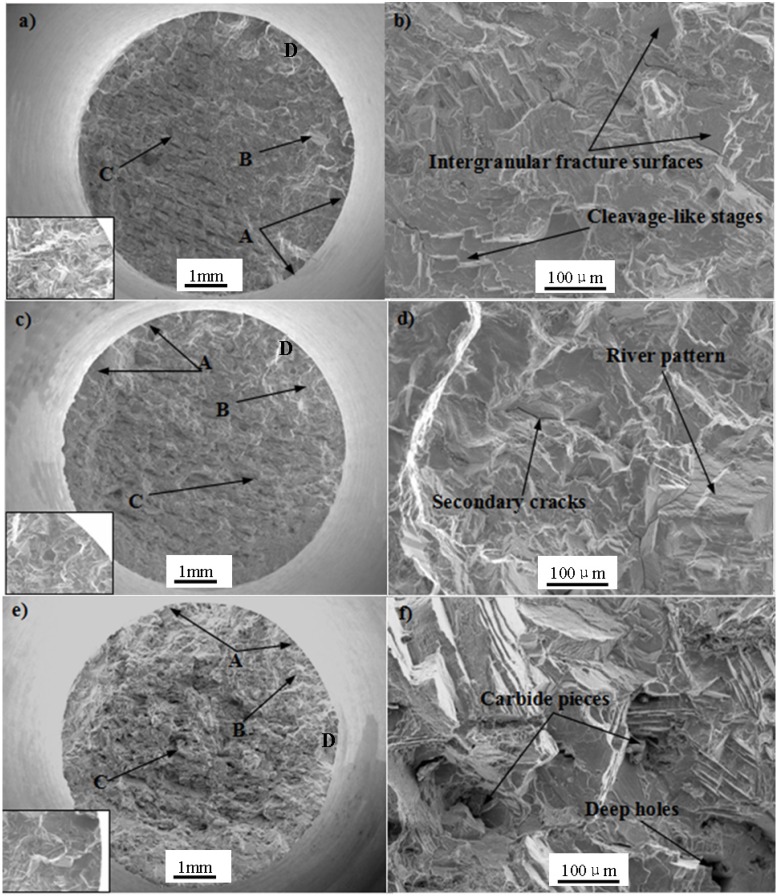
Fractographs of Pro.I after LCF at 650 °C. (**a**,**b**) Δε_t_/2 = 0.4%; (**c**,**d**) Δε_t_/2 = 0.6%; (**e**,**f**) Δε_t_/2 = 0.8%.

**Figure 8:**
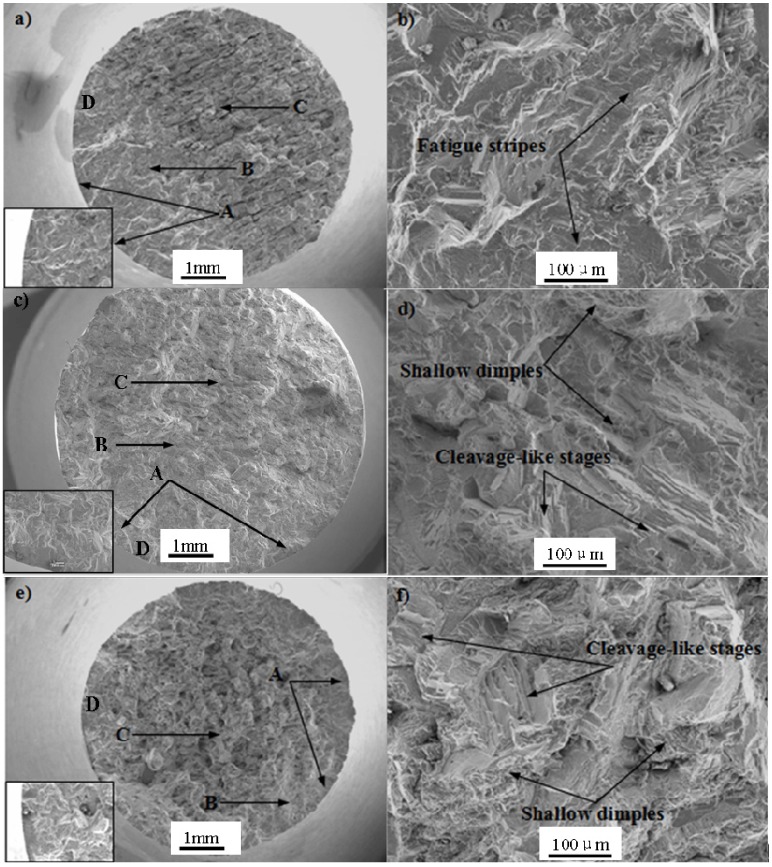
Fractographs of Pro.II after LCF at 650 °C. (**a**,**b**) Δε_t_/2 = 0.4%; (**c**,**d**) Δε_t_/2 = 0.6%; (**e**,**f**) Δε_t_/2 = 0.8%.

Typical fatigue morphology includes fatigue source region, crack propagation region and final instant rupture region. The macroscopic and microscopic fatigue fracture morphologies of Pro.I and Pro.II are shown in Figure 7 and Figure 8, respectively, where, Zone A: fatigue source region, Zone B: crack propagation region, Zone C: final instant rupture region, Zone D: the original locations for the figures of high magnification of crack initiation site located in bottom left in Figure 7a,c,e and Figure 8a,c,e. The pictures (b), (d) and (f) in Figure 7 and Figure 8 were taken near crack propagation region.

Pro.I and Pro.II have some similar macroscopic fracture morphologies. It can be seen from the macroscopic fracture morphologies that the final instant rupture region becomes gradually larger with the increase of strain amplitudes. Moreover, there are multiple fatigue sources for the fracture morphology of the alloy after LCF at 650 °C under different strain amplitudes. It can be seen that the fatigue cracks initiate at the site of discontinuous carbides at the grain boundary from the figures of high magnification of crack initiation site. The occurrence of carbides after heat treatment results in fragile boundaries. In addition, high temperature of 650°C leads to the reduction of the strength of grain boundary, where the fatigue cracks began to initiate at the grain boundary.

In terms of Pro.I, under total strain amplitude of 0.4%, intergranular fracture surfaces, secondary cracks and cleavage-like stages can be observed. The cleavage-like stage is composed of many cleavage planes. Under total strain amplitude of 0.6%, intergranular fracture surfaces, secondary cracks and river pattern can be discovered. Under total strain amplitude of 0.8%, intergranular fracture surfaces and deep holes can be found. A number of shallow dimples occur on some intergranular fracture surfaces. Carbide pieces exist in the deep holes.

In terms of Pro.II, under total strain amplitude of 0.4%, a large number of ductile fatigue stripes and a small amount of cleavage-like stages exist in fracture surface (Figure 8b). Moreover, a small amount of secondary cracks occur on the fracture surface which are parallel to fatigue stripes. Fatigue strips are caused by alternating plastic passivation opening and closing sharpening at crack tip. Under total strain amplitude of 0.6%, a small amount of the intergranular fracture and plenty of cleavage-like stages can be observed. In addition, a large number of shallow dimples can be observed. Under total strain amplitude of 0.8%, brittle dendritic fracture, a large number of cleavage-like stages and dimples can be found. Moreover, many carbide pieces exist on the fracture surface.

According to above observations, it can be concluded that the fatigue fracture mechanism of Pro.I and Pro.II during LCF process is the combined effects of brittle fracture and ductile fracture. The shallow dimples at the specimen surface indicate the occurrence of plastic deformation. The appearance of intergranular fracture surfaces and occurrence of cleavage-like stages related with the imposed strain range indicate the occurrence of brittle fracture. Hong *et al.* [16] claimed that the failure mechanism was essentially the crack initiation at the oxide-layered surface and its planar growth along <100> γ channel in LCF conditions. Yu *et al.* [17] thought that the fatigue failure mechanism was crack initiating along the slip bands that intersect the defects (micropores) along the deformed zone and shearing decohesion while the pore induces a local stress concentration. In this paper, the cracks initiate at multi-sites of free surface of the alloy (Figure 7a,c,e and Figure 8a,c,e). Then cracks propagate in some way perpendicular to the applied loading. Cracks will be subjected to obstruction when they propagate. The microscopic fatigue fracture morphologies show the interaction between dislocations and γ′ phase.

The intergranular fracture surfaces occur on the fatigue fracture morphologies of Pro.I and Pro.II, which shows that the alloy produces intergranular brittle fracture due to grain boundaries weakening. The reasons of grain boundaries weakening are as follows: (1) impurity elements pile up on grain boundary; (2) the precipitates pile up on grain boundary; (3) the erosion of environmental medium; (4) high temperature. In present study, intergranular brittle fracture is caused by reasons (2) and (4). The discontinuous carbides on the grain boundary after heat treatment result in fragile boundaries in the low cycle fatigue process. At the same time, high temperature leads to the reduction of the strength of grain boundary. Thus, the fatigue properties come to decline and the intergranular fractures occur.

### 3.5. Dislocation Structures and Deformation Mechanism

**Figure 9:**
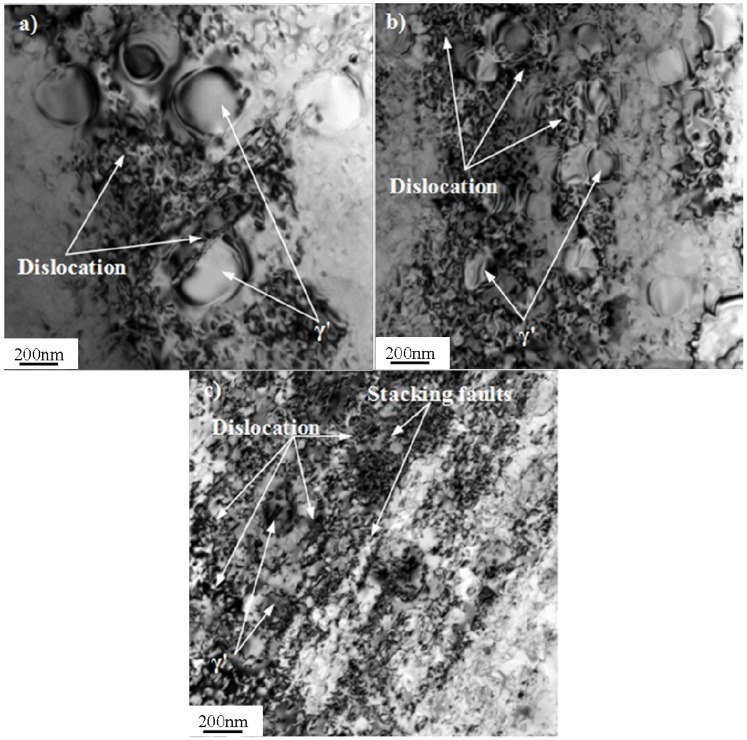
Typical dislocation structures of Pro.I after LCF tests at 650 °C (bright field). (**a**) Δε_t_/2 = 0.4%, pcum = 3.562; (**b**) Δε_t_/2 = 0.6%, pcum = 1.952; (**c**) Δε_t_/2 = 0.8%, pcum = 1.012.

**Figure 10:**
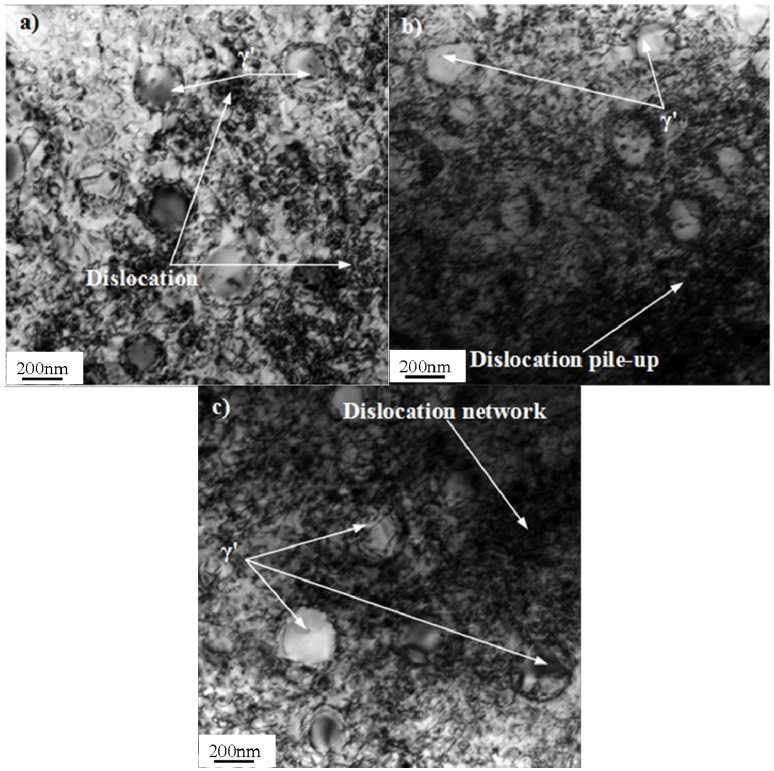
Typical dislocation structures of Pro.II after LCF tests at 650 °C (bright field). (**a**) Δε_t_/2 = 0.4%, pcum = 2.898; (**b**) Δε_t_/2 = 0.6%, pcum = 1.738; (**c**) Δε_t_/2 = 0.8%, pcum = 0.867.

Typical dislocation structures of Pro.I and Pro.II after LCF tests are shown in Figure 9 and Figure 10, respectively. The values of plastic deformation accumulated (pcum) during the test are also shown in Figure 9 and Figure 10.

In terms of Pro.I, under total strain amplitude of 0.4%, the distribution of dislocations is inhomogeneous. In some local regions dislocation cannot be observed. Dislocations are mainly distributed in the interface of γ' phase and the matrix phase. Dislocation pile-up is marked. Several parallel dislocation lines and different sizes of γ' phase can be found. It is obvious that dislocations cross through γ' phase (Figure 9a). Under middle strain amplitude of 0.6%, the number of dislocations significantly increases. Dislocation pile-up is more marked than some dislocation depleted regions (Figure 9b). Under total strain amplitude of 0.8%, dislocations present short shape. Most of the dislocations pile up on the boundary of γ' phase. A small portion of the dislocations cut into γ' phase (Figure 9c).

In terms of Pro.II, under total strain amplitude of 0.4%, dislocations tangle into a dislocation network. Dislocations generate interaction with γ' phases and dislocations cut into γ' phase (Figure 10a). Under middle strain amplitude of 0.6%, dislocation pile-up is more marked than some dislocation depleted regions. Most dislocations distribute in the interface of γ' phase and matrix phase. A small amount of dislocations cut into γ' phase (Figure 10b). Under total strain amplitude of 0.8%, the number of dislocations is relative the largest. The interaction of dislocations and γ' phases is most apparent. The locations of dislocation pile-up increase (Figure 10c).

Through analysis about dislocation structures, it is obvious that the deformation of the alloy during LCF process is inhomogeneous (Figure 9a). As mentioned above, cyclic stress response behaviors include cycle hardening, softening and stability. Cyclic hardening phenomenon is caused by proliferation of dislocation hampering dislocation movement (Figure 9b). It is also associated with the interactions between dislocations and strengthening phase (Figure 10b). The cyclic softening phenomenon is due to the dislocations cutting orderly γ' phase and causing disorder, which reduces the deformation resistance of the alloy (Figure 9a and Figure 10a). It can also be caused because the growth of γ' phase reduces strengthening effect in the LCF process. Moreover, the temperature of 650 °C is high enough to provoke an aging treatment. The mobile dislocation can be trapped into a growing precipitate resulting in softening phenomenon. The cyclic stability can result from a dynamic equilibrium between the hardening effect from proliferation of dislocation hampering reciprocating motion of dislocations and the softening effect from dislocations cutting orderly γ′ phase.

According to above observations, it can be concluded that the deformation mechanisms of Pro.I and Pro.II during LCF process are similar. The distributions of dislocation become more homogeneous with the increase of strain amplitude. Moreover, the density of dislocation increases and dislocation pile-up becomes more marked with the increase of strain amplitude. The formation of dislocation network is caused by proliferation of dislocations (Figure 9b and Figure 10b). The proliferation of dislocations and dislocation pile-up hinder further movement of dislocations (Figure 9c and Figure 10c). The interactions between dislocations and the strengthening phase present different forms. Some dislocations run across γ′ precipitates or cut into γ′ precipitates (Figure 9a and Figure 10a). Some dislocations bypass γ′ precipitates or bow out between γ′ precipitates and form dislocation loops around γ′ precipitates (Figure 10b). At lower strain levels, the precipitates are hard to deform and dislocations mainly pile up on the boundary of γ′ phase. At higher strain levels, the precipitates are easy to deform and dislocations mainly cut into γ′ phase. The precipitates are more prone to be cut by dislocation with increasing strain amplitude. At lower strain levels, a small number of dislocations show that the alloy produces less plastic deformation. Dislocation pile-up is hard to reach the degree of stress concentration. Thus, cracks begin to initiate after a large number of fatigue cycles. In this case, the alloy has a long fatigue life. At higher strain levels, a large number of dislocations show that the alloy produces larger plastic deformation. A large number of dislocation pile-up results in stress concentration, which leads to the occurrence of cracks initiation. Thus, the fatigue fracture of the alloy occurs in a short cycles. In this case, the alloy has a short fatigue life. It can be obtained from Figure 9 and Figure 10 that the values of plastic deformation accumulated (pcum) decrease with the increase of total strain amplitudes under the condition of the same heat treatment procedure. It is due to a large number of fatigue cycles at low total strain amplitude, though the small peak plastic amplitude, which is shown in Table 1. The proportion of the plastic strain in total strain gradually increases with increasing total strain. It increases from 1% to 36.25% for Pro.I and from 1% to 36.5% for Pro.II from the total amplitude of 0.3% to amplitude of 0.8%, which shows that plastic strain accounts for a small proportion in the total strain. It can be found that the increasing proportion of the plastic strain in total strain leads to the reduction of fatigue life for the alloy. It also indicates that the values of plastic deformation accumulated are associated with plastic amplitude. During the LCF process, the smaller plastic strain amplitude, the greater the values of plastic deformation accumulated, which represents the perfect fatigue resistance of the alloy. Moreover, the value of plastic deformation accumulated for Pro.I is larger than that for Pro.II at same total strain amplitude. It indicates that the nickel-base superalloy possesses more excellent fatigue resistance through the heat treatment of Pro.I than that of Pro.II.

## 4. Conclusions

In this paper, two kinds of heat treatment procedures were undertaken to investigate the effect of heat treatment on microstructure and LCF behaviors of a nickel-base superalloy. With the wide application of nickel-base superalloys, research on the fatigue behaviors of hot-end components such as turbine disks have become a research hotspot. In actual service process, improvement in fatigue performance is of great significance. Proper heat treatment processes significantly improv the mechanical properties of superalloys especially fatigue resistance through changing the internal organizational structure. Thus, hot-end components with perfect fatigue performance can serve safely and reliably. It is meaningful to improve the production and application of nickel-base superalloys as well as the life of hot-end components through investigating the effects of heat treatment process on LCF behaviors. The main results drawn from this study are as follows:

(1) The heat treatment process plays an important role in microstructure and fatigue behavior. The fatigue resistance of Pro.I and Pro.II significantly drops with the increase of strain amplitude. The value of plastic deformation accumulated for Pro.I is larger than that for Pro.II at same total strain amplitude, which shows that the fatigue resistance performance of Pro.I is better than that of Pro.II.

(2) The predicted effect of Manson-Coffin relationship is more accurate than that of three-parameter power function for high total strain amplitude section. On the contrary, the predicted effect of three-parameter power function is more accurate than that of Manson-Coffin relationship for low total strain amplitude section.

(3) The fatigue fracture mechanism of Pro.I and Pro.II during LCF process is the combined effects of brittle fracture and ductile fracture. The main deformation mode of the alloy is inhomogeneous deformation and the different interactions between dislocations and γ′ phases.
